# Nursing-engineering interdisciplinary research: A synthesis of methodological approach to perform healthcare-technology integrated projects

**DOI:** 10.1016/j.mex.2023.102525

**Published:** 2023-12-16

**Authors:** Marco Sguanci, Stefano Mancin, Michela Piredda, Francesca Cordella, Nevio Luigi Tagliamonte, Loredana Zollo, Maria Grazia De Marinis

**Affiliations:** a Department of Medicine and Surgery, Research Unit of Nursing Science, Università Campus Bio-Medico di Roma, Via Alvaro del Portillo, 21 - 00128 Roma, Italy; b Department of Biomedical Sciences, Humanitas University, 20090, Milan; c IRCCS Humanitas Research Hospital via Manzoni, 56 20089 Rozzano - Milan, Italy; d Department of Biomedicine and Prevention, University of Rome “Tor Vergata”, Viale Montpellier, 1- 00128 Rome, Italy; e Unit of Advanced Robotics and Human-Centred Technologies, Università Campus Bio-Medico Via Alvaro del Portillo, 21, 00128, Roma, Italy; f Fondazione Policlinico Universitario Campus Bio-Medico, Via Alvaro del Portillo 200 - 00128, Roma, Italy

**Keywords:** Healthcare, Nursing, Engineering, Methodology, Guidelines, Quality of life improvement, Technology, Healthcare project, Interdiscipinary, Narrative review

## Abstract

In the dynamic landscape of contemporary healthcare, the imperative for advancing the frontiers of knowledge and improving patient outcomes necessitates a paradigm shift towards a multidisciplinary approach. This background great enhances a nurse's ability to interface with technology and create technical solutions such as robots, patient care devices, or computer simulation for patient care needs and nursing care delivery. This study aims to describe, through a narrative review of evidence, a methodology to develop and manager Nursing-Engineering interdisciplinary project, clarify the key points and facilitate professionals who are not very familiar with this topic. The methodology employed highlights the importance of this kind of research that allows to achieve highest standards of practice leading to improved patient care, innovative solutions and a global contribution to healthcare excellence.

## Review Highlights

• Supply a structured framework that guides the development of interdisciplinary projects in nursing and biomedical engineering**.**
• Encourage researchers to engage in interdisciplinary collaboration that allows for the incorporation of diverse expertise, leading to more comprehensive and innovative projects.
• Highlights the profound importance of interdisciplinary projects in healthcare, nursing, biomedical engineering, and robotics and its capacity to drives innovation, and broadens the horizons of scientific inquiry to elevate the standards of patient care

## Graphical abstract

**Figure:**
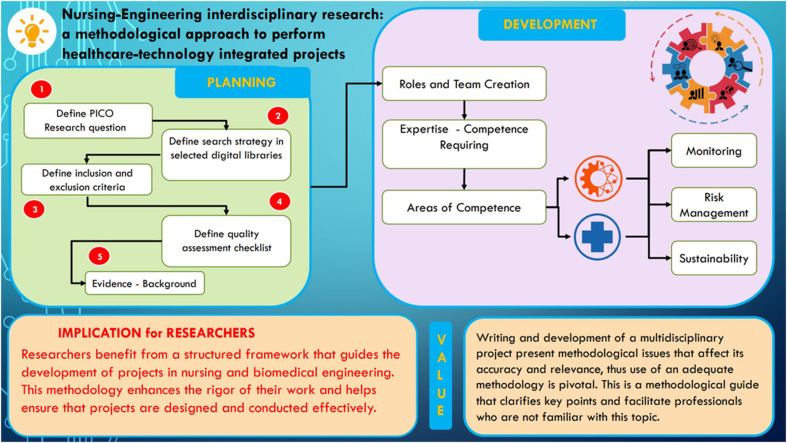

Specifications table

**Table:** 

Subject area:	Medicine and Dentistry
More specific subject area:	Interdisciplinary Research Projects
Name of the reviewed methodology:	Narrative review of methodology in development and management of heathcare-technology integrated project
Keywords:	Healthcare, nursing, engineering, methodology, guidelines, quality of life improvement, technology, healthcare project, interdisciplinary
Resource availability:	N/A
Review question:	• How to perform bioengineering-nursing project? • How do you define the most important steps in designing interdisciplinary research?

## Method details

### Background and research methodology objective

In the dynamic landscape of contemporary healthcare, the imperative for advancing the frontiers of knowledge and improving patient outcomes necessitates a paradigm shift towards a multidisciplinary approach.

Interdisciplinary research or project (IR or IP) encompasses the collaborative efforts of two or more academic disciplines to explore and define the nature of learning. Strober in 2011 [1] referred to related concepts like cross-disciplinary, interdisciplinary, and transdisciplinary. These terms are often used interchangeably, indicating a shared goal of bridging the gaps between various academic areas, especially in research inquiries and methodological approaches. Interdisciplinarity strives for a deeper fusion of methodologies, theories, content, and perspectives and promotes interactions that give rise to fresh research themes, novel concepts, and an expansion and deepening of research inquiries.

Transdisciplinary, in contrast, signifies an even higher level of integration, involving the transfer and transcending of knowledge and research, going beyond the confines of individual disciplines [2].

This kind of research has become increasingly prominent since the mid-20th century standing as a fundamental point in healthcare, encompassing various scientific disciplines and fostering a holistic perspective. Nowhere is the significance of this interdisciplinary research paradigm more pronounced than in the realm of healthcare, nursing, biomedical engineering, and robotics, where the confluence of diverse scientific fields, from biology and medicine to psychology, sociology, engineering, and artificial intelligence, converges to offer a comprehensive understanding of health and the human condition.

In an era that expects multidisciplinary education and practice, nursing collaborations with less traditional professional partners, including engineers, are becoming increasingly important [3].

This background would greatly enhance a nurse's ability to interface with technology and create technical solutions such as robots, patient care devices, or computer simulation for patient care needs and nursing care delivery [3]. Conversely, a healthcare background with requisite clinical experience would greatly enhance a biomedical engineer's ability to solve clinical problems.

This particular scientific approach merges the principles of these different sectors propelling technological innovations and enhancing our comprehension of human physiology and disease mechanisms.

Huston et al. (2013) [4] said that the greatest challenges for nurses include the integration of new technologies into practice and managing the human technology interface: in this view, the integration of robotics in nursing and healthcare offers the potential for improved efficiency and precision in various healthcare settings.

This highlights the profound importance of IP in healthcare, nursing, biomedical engineering, and robotics, unveiling the myriad ways in which it enhances the quality of care, drives innovation, and broadens the horizons of scientific inquiry to elevate the standards of patient well-being as calling by international governmental agencies like the Agency for Healthcare Research and Quality [5] and organizations such as the Robert Wood Johnson Foundation [6].

The development of healthcare-technology (Health-Tech) can be defined as a knowledge search by an objective and systematic method for an original contribution to the existing stock of knowledge involving a combination of several scientific disciplines and methods: this study aims to describe a methodological approach to perform a interdisciplinary nursing-engineering project applicable to all IP in healthcare nurse-engineering setting.

## Methods

The literature screening shown important information: one of this is perform a clear and simple methodology. Interdisciplinary project development will be divided into two main phases (Planning and Development) in which are described the specific activities.

## Planning

### Formulation of the research question

The general research question for interdisciplinary projects was developed using the PICO model [7].

The PICO process (or framework) is a research tool used in evidence-based practice (and specifically evidence-based medicine) to frame and answer a clinical or health care related question though it is also argued that PICO "can be used universally for every scientific endeavour in any discipline with all study designs.

The PICO acronym usually has come to stand for: **P** (Patient, problem or population; **I** (Intervention); **C** (Comparison, control or comparator); **O** (Outcome(s)).

Three main aspects (PIO) were included in this review: *P* = patients with an objective or subjective criticality; *I* = interdisciplinary interventions.; *O*= describe the outcomes resulting from the adoption of a specific technology in terms of impact in patient's quality of life.

### Review of evidence

A primary fundamental point in embarking upon a interdisciplinary healthcare project lies in the comprehensive review of existing scientific literature: the synthesis of knowledge from diverse fields, such as medicine, nursing, biomedical engineering, and related areas, can provide a critical foundation for the design and implementation of innovative healthcare initiatives. A thorough review of scientific literature not only acquaints researchers and practitioners with the current state of knowledge but also identifies gaps and opportunities where the integration of multidisciplinary expertise can yield transformative results. This foundational step not only informs the conceptualization of the project but also ensures that it is rooted in evidence-based practices, promoting the highest standards of care and research integrity. The search strategy must be performed using the key terms defined in the research problem through consultation of the scientific databases relevant for healthcare and biomedical engineering (PubMed/Medline, Embase, CINAHL, Scopus, Cochrane Library, Web of Science). The search must be exhaustive and therefore follow the methodology of systematic reviews according to the Preferred Reporting Items for a Systematic Review and Meta-analysis [8].

### Purpose definition

In the context of a joint bioengineering and nursing project aimed at improving patient health through the implementation of technology, the definition of primary and secondary objectives is crucial for a clear delineation of the project's aims and goals. Primary objectives focus on the primary goal of enhancing patient health through the proposed technology: they must be defined starting from the clinical field explored (i.e.: mobility, rehabilitation, quality of life, self-management in chronic disease, telemedicine, education, home care, support of healthcare professionals) [9].

The objectives must be clear shared by the team as underlined by Social Care Institute for Excellent [10].

Objective must be [11]:

• Specific (S): Objectives should be precise and clearly defined, leaving no room for ambiguity. They must focus on a particular aspect of healthcare or a specific problem to address.
• Measurable (M): Objectives should be quantifiable, allowing for the assessment of progress and success. This often involves using metrics or indicators to track outcomes.
• Achievable (A): Objectives must be realistic and attainable given the available resources, expertise, and constraints. They should challenge the team without being overly ambitious.
• Relevant (R): Objectives should align with the overall goals of the healthcare project and the specific needs of the target patient population.
• Time-Bound (T): Objectives should have a clearly defined timeframe or deadline for completion.

In case of interdisciplinary project, the objective is also be:

• Interdisciplinary (I): In interdisciplinary healthcare projects, objectives should require collaboration and input from various fields such as medicine, nursing, engineering, and others. They should leverage the strengths of each discipline.
• Patient-Centered (P): Objectives should ultimately benefit the patient. They should aim to improve patient outcomes, experiences, or the quality of care provided.
• Innovative (I): Objectives should encourage creative thinking and the development of novel solutions. They should push the boundaries of existing knowledge and practices.
• Evidence-Based (E): Objectives should be grounded in scientific research and empirical evidence, ensuring that proposed interventions are likely to be effective and safe.
• Ethical (E): Objectives should adhere to ethical principles and guidelines in healthcare, prioritizing patient well-being, safety, and consent.

### Outcome definition

The definition of primary and secondary outcomes is paramount when assessing the impact of technological innovations on patient well-being. Primary outcomes represent the core objectives of the project, often directly related to the primary goal of enhancing patient care.

These might include measures such as:

• the reduction of pain
• improvement in quality of life
• increase in the efficacy of a healthcare procedure or device through the implementation of new technologies

Secondary outcomes encompass a broader spectrum of parameters that, can offer valuable insights into the holistic benefits of the technology. Secondary outcomes may include factors like:

• patient satisfaction
• cost-effectiveness analysis
• potential long-term impact of the innovation on overall health and healthcare delivery
• sustainability

This definition process refers to the historic model of Donabedian [12] that includes the factors:

• the team composition
• the number of disciplines working together
• the patient population
• the number of sectors included in the collaboration

**Fig. 1:**
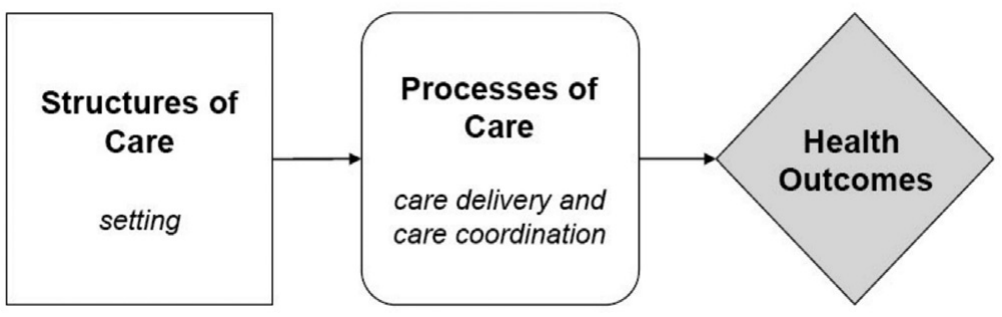
Donabedian quality framework [13].

In his model, Donabedian looks at ‘outcomes’ as the effects of health care on patients or populations, including changes in health status (clinical): the precise delineation of these outcomes ensures that the impact of the technology is comprehensively assessed, allowing for a more informed, evidence-based approach to the integration of bioengineering and nursing in patient care: defining and measuring these outcomes with precision, projects in this domain can ascertain not only the effectiveness but also the broader implications of the technology, ultimately improving patient outcomes and healthcare as a whole (Fig. 1).

### Indicator definition

Indicators development for interdisciplinary projects in nursing and biomedical engineering is an ongoing process that requires attention to detail, collaboration, and a commitment to improving patient care and healthcare practices.

**Fig. 2:**
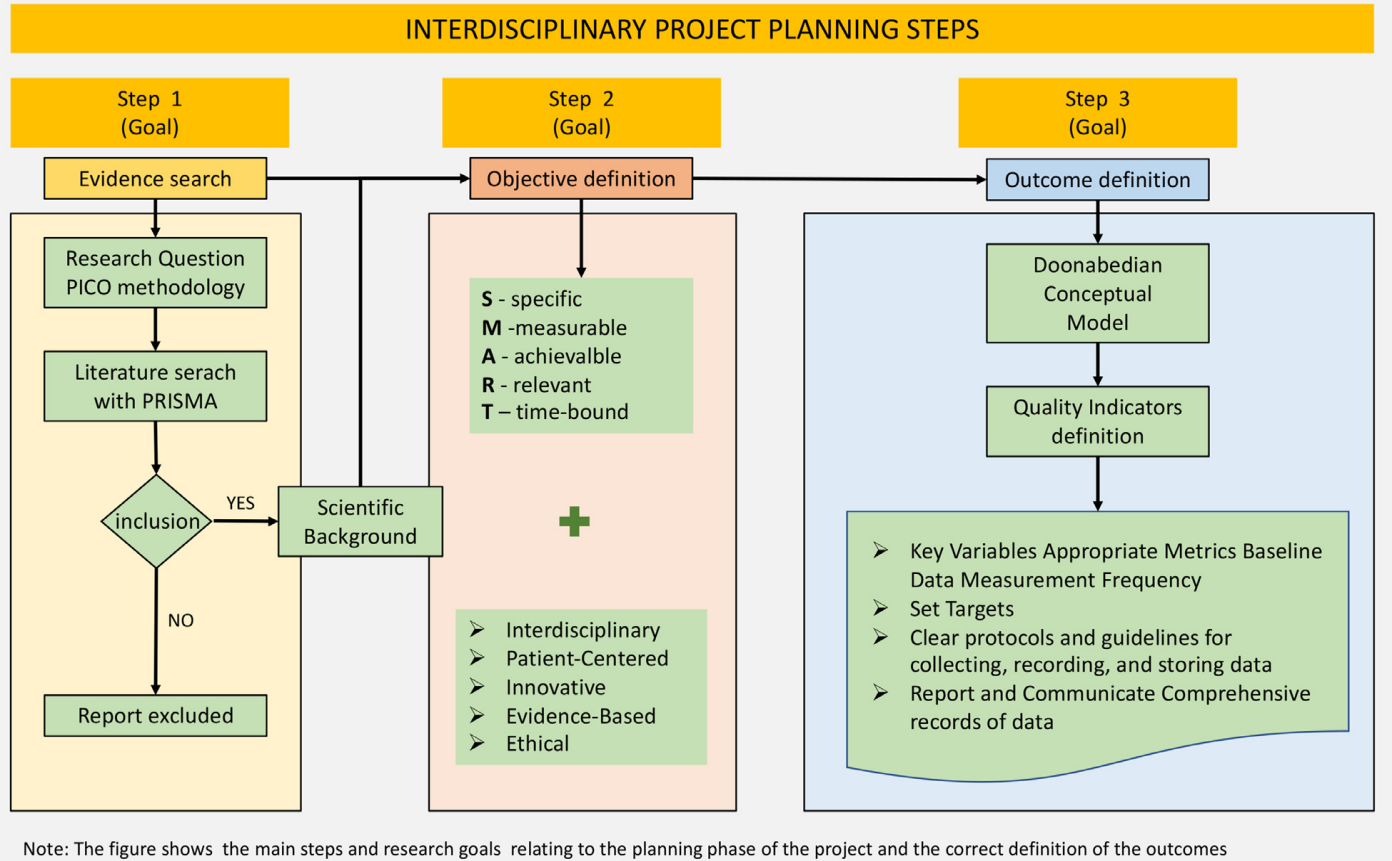
Flowchart project planning.

To develop a project clearly it is necessary that the indicators chosen fall into three main categories: outcome, structure and process according to Donabedian model, still current reference from 1980 for interdisciplinary projects [14]. Fig. 2 summarize the planning step.

The main necessary steps are:

• Identify Key Variables: Identify the critical variables or factors that are directly related to your project's objectives. These variables can be qualitative or quantitative and should be measurable.
• Select Appropriate Metrics: Choose the most suitable metrics or measurement tools to quantify the identified variables. This may include surveys, medical devices, data collection tools, or other assessment methods.
• Establish Baseline Data: If applicable, collect baseline data to understand the current state of the variables before implementing your project as a reference point for measuring improvements.
• Determine Measurement Frequency: Decide how frequently you will measure the chosen variables.
• Set Targets: Define target values or benchmarks for each indicator.
• Create clear protocols and guidelines for collecting, recording, and storing data
• Report and Communicate: Share your findings and progress with the multidisciplinary team, stakeholders, and relevant parties in a clear and transparent manner. Effective communication is vital for collaboration.
• Create a comprehensive record of all data, findings, and changes made during the project. This documentation will be crucial for future reference and knowledge sharing.

### Development

#### Roles of partners and team definition

**Fig. 3:**
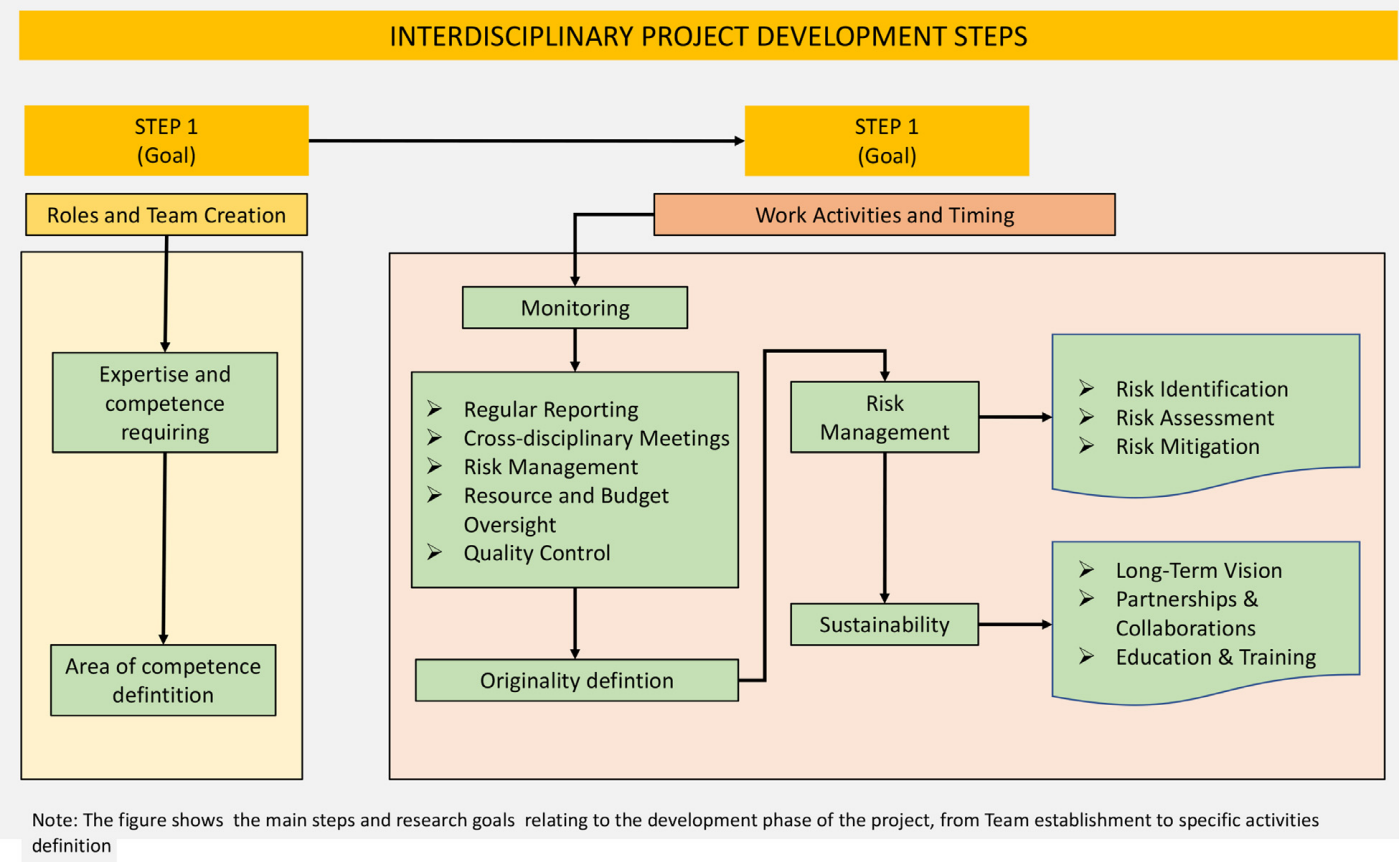
Flowchart project development.

Establishing a interdisciplinary research demands a thoughtful approach that emphasizes effective collaboration, a shared commitment to healthcare innovation (Fig. 3).

In nursing-engineering project this is fundamental as these projects develop new technologies in different clinical contexts (hospitals, homes); clinical skills allow you to develop technical ideas by contextualizing the approach and technical skills allow to materialize them.

In the first step it is mandatory determine required expertise and recruit team members from diverse backgrounds who possess the necessary skills and knowledge.

In second step it is necessary compose a team with a mix of roles: in Health-Tech project this means include: nurses, biomedical engineers, data scientists, clinicians and project managers. Subsequently need to define clear roles and responsibilities for each team member establishing how they will collaborate and contribute to the project's success.

The following table summarize common areas of competence (Table 1).

**Table 1:** Areas of competence.

Step	Technical area (Engineering)	Clinical area (Nursing)
Determine Required Expertise	Identify the necessary areas of engineering expertise for the project	Identify the necessary areas of healthcare expertise for the project
Recruitment and Selection	Recruit engineering team members with relevant skills in biomedical engineering, data science, and technology development	Recruit clinical team members with expertise in nursing, medicine, and patient care.
Team Composition	Compose an engineering team with roles including biomedical engineers, data scientists, and other technical specialists.	Compose a clinical team with roles including nurses, clinicians, and other healthcare professionals.
Ethical Considerations		Address ethical concerns related to patient care, privacy, informed consent, and adherence to ethical guidelines.
Data Management		Establish data management protocols to ensure secure and ethical handling of patient data and research findings.
Resource Allocation	Allocate engineering resources, including funding and specialized equipment, to support the technical aspects of the project.	
Dissemination and Publication	Promote the sharing of research findings with a focus on technical conferences, publications, and clinical collaborations	Promote the sharing of research findings with a focus on healthcare conferences, publications, and clinical collaborations.
Sustainability	Plan for the sustainability of the clinical team and research in terms of long-term cost-effective of new technology	Plan for the sustainability of the clinical team and research in terms of long-term patient care and collaborations with healthcare institutions.

### Work activities and timing

For a correct management of activities, it is necessary to identify them in development containers called Work Packages (WP): it is a fundamental component of project management, particularly in complex or interdisciplinary projects. It represents a division of project tasks into specific, manageable units. Each work package must define: objectives, responsibilities, resources and timing. The primary purpose of this approach is to streamline the planning, execution, and control of activities.

Usually WP they must always contain key information such as:

• Objective Definition
• WP Manger
• Resources (personnel, budget, equipment, and other elements, to successfully complete the WP)
• Activity Planning
• Timing
• Budgeting
• Control and Monitoring (control criteria to assess the progress of WP)
• Communication: (inform about the status of WP) [15]
• Documentation: (record all information related to the work package
• Evaluation: (assess the achievement of WP objectives)

### Originality definition

To propose a Health-Tech project it is recommended to focus attention on the originality of the idea and its potential development.

Interdisciplinary project stands out for its ability to generate innovative solutions through interdisciplinary collaboration, thus bringing new insights and methodologies to address relevant healthcare challenges.

The topic of this section must describe the impact for clinical practice and quality of care in terms of outcomes for patients and underlining that emerging findings could be adopted globally, significantly contributing to healthcare innovation.

Highlight also that interaction between biomedical engineers and nursing professionals can lead to solutions that positively impact people's lives and the way medical care is delivered.

### Activity monitoring

Monitoring strategies in a IP can maintain alignment, ensure progress, and achieve its objectives effectively and efficiently as described in Table 2.

**Table 2:** Activity monitoring.

Aspect	Description
Regular Reporting	Continuously track progress through status reports from team members in each discipline.
Cross-disciplinary Meetings	Hold regular meetings to ensure alignment, share updates, and discuss challenges and solutions. [16]
Risk Management	Identify, assess, and mitigate risks that may affect project progress.
Resource and Budget Oversight	Monitor resource allocation and budget utilization for efficient management.
Quality Control	Implement quality control processes to maintain project standards, meet criteria, and seek improvements.

### Risk management and sustainability

Risk management is mandatory in interdisciplinary projects: it serves to identify, assess, and mitigate potential challenges and uncertainties that could disrupt the project's success. In a project involving various disciplines, such as biomedical and nursing, it is crucial for interdisciplinary integration, continuity and resource optimization.

Sustainability in a Health-Tech projects extends the impact beyond its immediate timeframe and it is decisive for long-term benefits, knowledge dissemination, compliance and growth. The table summarize this main aspects (Table 3).

**Table 3:** Risk management activities and sustainability.

*Risk Management*	
Aspect	Description
Risk Identification	Collaboratively identify and document potential risks, considering technical and clinical aspects.
Risk Assessment	Evaluate the likelihood and impact of risks, prioritizing them based on severity and potential consequences.
Risk Mitigation	Develop strategies to mitigate or manage identified risks, with a focus on interdisciplinary solutions.

*Sustainability*	
Aspect	Description
Long-Term Vision	Define a long-term vision for the project's outcomes, emphasizing post-project healthcare benefits.
Partnerships & Collaborations	Establish lasting partnerships to ensure continued adoption and integration of project results
Education & Training	Develop ongoing educational and training programs to empower healthcare professionals in utilizing project outcomes effectively.

For these reasons it is necessary to include a dedicated section in the projects that clarifies the management policies and activities that are intended to be adopted.

## Discussion and implication for researchers

This article, based on the evidence available in the literature which has proven to be very limited, aimed to summarize the current knowledge on this specific topic. Interdisciplinary projects are still little discussed and bibliographical sources provide generic indications. From our experience and from the scientific material found in this review we can however provide some implications that our study may have for the development of future projects.

In order to conduct an interdisciplinary project in the correct way we suggest to apply:

• *A guided methodology*: Researchers benefit from a structured framework that guides the development of projects in nursing and biomedical engineering. This methodology enhances the rigor of their work and helps ensure that projects are designed and conducted effectively.
• *An efficacy interdisciplinary collaboration*: Researchers are encouraged to engage in interdisciplinary collaboration. This not only enriches the research process but also allows for the incorporation of diverse perspectives and expertise, leading to more comprehensive and innovative projects.
• *A publication and dissemination policy*: Methodological articles can serve as a valuable resource for researchers seeking to publish their work. They provide insights into the best practices for project development, which can enhance the quality and impact of research publications.
• *A professional growth plan*: Researchers can use this methodological approach to refine their skills and knowledge in project development. This contributes to their professional growth and competence in the field of nursing and biomedical engineering.
• *A continuous project improvement*: Researchers participating in IP are motivated to embrace best practices and continually improve their project development processes benefiting the broader fields of nursing and biomedical engineering.

## Conclusion

The writing and development of interdisciplinary project, especially in nursing-engineering area, due to its complex nature, it may present important methodological obstacles that could affect its accuracy and relevance.

An adequate methodology allows for correct management of resources and competencies, encouraging comparison between different disciplines and enhancing the skills of the participants.

In projects that involve the interaction of two very different worlds such as clinical and technology, the methodological approach is even more fundamental.

We decided to perform a methodological guide for professionals (clinical or technician), contextualized in nursing-engineering area, to clarify the key points and facilitate professionals who are not very familiar with this topic.

This kind of projects are a testament to the commitment of these disciplines to the highest standards of practice and research, ultimately leading to improved patient care, innovative solutions, and a global contribution to healthcare excellence.

## Ethics statements

No ethical approval was required for the purposes of this study, as it is unnecessary.

## Funding

This research did not receive any specific grant from funding agencies in the public, commercial, or not-for-profit sectors.

## CRediT authorship contribution statement

**Marco Sguanci:** Conceptualization, Methodology, Writing – original draft, Writing – review & editing, Investigation, Visualization. **Stefano Mancin:** Conceptualization, Methodology, Writing – original draft, Writing – review & editing, Investigation, Visualization. **Michela Piredda:** Methodology, Investigation, Writing – review & editing, Visualization. **Francesca Cordella:** Conceptualization, Methodology, Writing – review & editing. **Nevio Luigi Tagliamonte:** Conceptualization, Methodology, Writing – review & editing. **Loredana Zollo:** Writing – review & editing. **Maria Grazia De Marinis:** Methodology, Writing – review & editing, Investigation, Visualization.

## Declaration of Competing Interest

The authors declare that they have no known competing financial interests or personal relationships that could have appeared to influence the work reported in this paper.

## Data Availability

Data will be made available on request.
